# Clinicopathologic characteristics, treatment, and outcomes of tubulointerstitial nephritis and uveitis syndrome in adults: A national retrospective strobe-compliant study

**DOI:** 10.1097/MD.0000000000003964

**Published:** 2016-07-01

**Authors:** Mathieu Legendre, Hervé Devilliers, Laurent Perard, Matthieu Groh, Habdelamid Nefti, Bertrand Dussol, Salim Trad, Fatouma Touré, Sébastien Abad, Jean-Jacques Boffa, Luc Frimat, Stéphane Torner, Alexandre Seidowsky, Ziad André Massy, David Saadoun, Virginie Rieu, Yoland Schoindre, Emmanuel Heron, Thierry Frouget, Arnaud Lionet, François Glowacki, Laurent Arnaud, Christiane Mousson, Jean-François Besancenot, Jean-Michel Rebibou, Philip Bielefeld

**Affiliations:** a Nephrology Department, Bocage University Hospital and UMR 1098; b Internal Medicine and Systemic Diseases Department, Bocage University Hospital, Dijon; c Internal Medicine Department, Edouard Herriot University Hospital and University Claude Bernard Lyon 1, Lyon; d Internal Medicine Department, National Referral Center for Rare Autoimmune and Systemic Diseases, Cochin University Hospital and University Paris Descartes, Paris; e Nephrology Department, Macon Hospital, Mâcon; f Nephrology Department, La Conception University Hospital and University Aix-Marseille, Marseille; g Internal Medicine Department, Ambroise-Paré University Hospital, Paris; h Nephrology Department, Maison Blanche University Hospital, Reims; i Internal Medicine Department, University Hospital Avicenne and University Sorbonne, Paris; j Nephrology Department, Tenon University Hospital, Paris; k Nephrology Department, Brabois University Hospital and INSERM CIC-EC CIE6, Nancy; l Nephrology Department, Dole Hospital, Dôle; m Nephrology Department, Ambroise-Paré University Hospital, Boulogne-Billancourt; n Internal Medicine and Clinical Immunology Department, Pitié Salpetriere University Hospital and DHU Inflammation, Immunopathology, Biotherapy, Paris VI; o Internal Medicine Department, University Hospital Clermont Ferrand, Clermont-Ferrand; p Internal Medicine Department, Quinze-Vingts Ophthalmogy Hospital, Paris; q Nephrology Department, University Hospital, Rennes; r Nephrology Department, University Hospital Claude Huriez, Lille; s Rheumatology Department, National Referral Center for Rare Autoimmune Diseases, Strasbourg University Hospital, and UMR 1109, Strasbourg, France.

**Keywords:** outcome, treatment, tubulointerstitial nephritis and uveitis syndrome

## Abstract

Tubulointerstitial nephritis and uveitis (TINU) syndrome is a rare disease, defined by the association of idiopathic acute TINU. The aim of our work was to determine the characteristics of adult TINU syndrome in France, and to assess factors (including treatment) influencing medium-term prognosis.

We conducted a nationwide study including 20 French hospitals. Clinical, laboratory, and renal histopathologic data of 41 biopsy-proven TINU syndromes were retrospectively collected. The patients were diagnosed between January 1, 1999 and December 1, 2015.

Twenty-five females and 16 males were included (F/M ratio: 1.6:1). The median age at disease onset was 46.8 years (range 16.8–77.4) with a median serum creatinine level at 207 μmol/L (range 100–1687) and a median estimated glomerular filtration rate (eGFR) at 27 mL/min per 1.73 m^2^ (range 2–73). Twenty-nine patients (71%) had a bilateral anterior uveitis and 24 (59%) had deterioration in general health at presentation. Moderate proteinuria was found in 32 patients (78%) (median proteinuria 0.52 g/24 h; range 0.10–2.10), aseptic leukocyturia in 25/36 patients (70%). The evaluation of renal biopsies revealed 41 patients (100%) with an acute tubulointerstitial nephritis, 19/39 patients (49%) with light to moderate fibrosis and 5 patients (12%) with an acute tubular necrosis. Thirty-six patients (88%) were treated with oral corticosteroids. After 1 year of follow-up, the median eGFR was 76 mL/min per 1.73 m^2^ (range 17–119) and 32% of the patients suffered from moderate to severe chronic kidney disease. Serum creatinine (*P* < 0.001, r = −0.54), serum bicarbonate and phosphate levels (respectively, *P* = 0.01, r = 0.53; and *P* = 0.04, r = 0.46), and age (*P* = 0.03, r = −0.37) at the 1st symptoms were associated with eGFR after 1 year. During the 1st year 40% of patients had uveitis relapses. The use of oral corticosteroids was not associated with a better kidney function but was associated with fewer uveitis relapses (*P* = 0.44 and 0.02, respectively).

In our study, 32% of patients were suffering from moderate to severe chronic kidney disease after 1 year of follow-up, and 40% had uveitis relapses during this follow-up. This work also suggests that oral corticosteroids are effective for the treatment of TINU syndrome's uveitis.

## 1 Introduction

Tubulointerstitial nephritis and uveitis (TINU) syndrome is a rare disease, defined by the association of idiopathic acute tubulointerstitial nephritis with uveitis.^[1,2]^ This syndrome is mostly described in children (with a predominance of females).^[2–4]^ Uveitis is typically anterior, bilateral but many atypical forms, notably with posterior uveitis, have been described.^[4–7]^ TINU syndrome is frequently accompanied by general signs, notably a deterioration in general health, weight loss, fever, and digestive symptoms.^[2,8,9]^ The pathogenesis of TINU syndrome is still unclear. It could appear in genetically predisposed patients (role of Human Leukocyte Antigen DRB1^∗^0102, DQA1^∗^01, DQB1^∗^05, and DQB1^∗^01),^[2,3,5,8]^ and could be triggered by medication, or the ingestion of toxic substances (i.e., the Chinese herb Goreisan, paracetamol, codeine phosphate, antibiotics, and nonsteroidal anti-inflammatory drugs)^[8,10,11]^ or by infections (i.e., Epstein–Barr virus, HTLV-1 virus, Chlamydia, herpes zoster virus).^[2,8,11–13]^ This syndrome is also frequently associated with other auto-immune diseases.^[2,8]^ Modified C-reactive protein (mCRP) could be the common uveal and renal antigen targeted by humoral and cellular auto-immune responses.^[8,14,15]^

Since the 1st descriptions by Dobrin et al in 1975, fewer than 170 adult cases have been published (around 40% of published cases of TINU syndrome), and most were isolated cases or small case series.^[15–18]^ Therapeutic management frequently comprises systemic and/or topical corticotherapy,^[2,15,19,20]^ although its efficacy has never been assessed in a comparative study with a control group. The renal prognosis of TINU syndrome is a matter of debate. Although TINU syndrome was long regarded as having a favorable renal prognosis,^[2,15,21,22]^ Li et al^[15]^ recently published contradictory data demonstrating that among patients of Chinese origin, renal recovery was incomplete in 92% of cases, 1 year after treatment onset. We present here what is to our knowledge the largest retrospective cooperative series of adult patients with TINU syndrome (41 patients).

We conducted the current study to provide a better description of clinical, laboratory, and histologic features at presentation; to determine whether recent data regarding renal prognosis of TINU syndrome were applicable to a French population; and to evaluate patients’ responses to corticosteroid therapy.

## 2 Methods

We performed a nationwide retrospective multicenter study in 20 French departments of nephrology, internal medicine, and ophthalmology. Patients were identified thank to a collaborative network: “le Club de Médecine Interne et Oeil (CMIO).” This study was conducted in compliance with the principles of the Declaration of Helsinki and its further amendments, and was approved by local institutional review board.

Included patients were diagnosed with TINU syndrome between January 1, 1999 and December 1, 2015. Patients met the following criteria: at least 1 attack corresponding to a «definite» or «probable» diagnosis of TINU syndrome according to the criteria of Mandeville et al,^[2]^ histology of the kidney puncture compatible with the diagnosis of TINU syndrome, were from mainland France and were older than 16 years at the time of the 1st symptoms, and exclusion of any alternative diagnosis.

Among 46 identified patients, 5 patients were not included due to the presence of mediastinal lymphadenopathy or unexplained hypercalcemia or elevation of angiotensin-converting enzyme or granuloma of the accessory salivary glands. All outcomes were established on December 1, 2015. All data were collected in each center by 1 author with a standardized form and were systematically reviewed by the same person.

### 2.1 Clinical and laboratory data at presentation

Data regarding history, clinical, ophthalmic angiography, and biological findings were collected for all patients. The World Health Organization criteria were used to define hypertension. Laboratory parameters collected were serum creatinine level (SCrl) (n = 41), CRP (n = 41), kalemia (n = 25), blood hemoglobin level (n = 40), eosinophilia (defined as >800.10^3^/mm^3^), serum calcium level (n = 31), phosphoremia (n = 29), plasma bicarbonate level (n = 26), albuminemia (n = 26), gammaglobulinemia (n = 35), proteinuria (n = 41), hematuria (defined as >10^4^ red blood cells/mL) (n = 41), leukocyturia (defined as >10^4^ leukocytes/mL) (n = 36), and glycosuria (n = 41). The estimated glomerular filtration rate (eGFR) was calculated using the simplified modification of diet in renal disease (MDRD) formula.^[23]^ Hypophosphatemia was defined as phosphoremia <0.80 mmol/L and hypokalemia was defined as kalemia <3.5 mmol/L. Fanconi syndrome was defined as the association of normoglycemic glycosuria, high phosphaturia and hypophosphatemia, amino aciduria, and type 2 renal tubular acidosis.^[24]^

### 2.2 Pathology analysis

Renal biopsies were analyzed in each center by a pathologist specialized in kidney histology. For light microscopic evaluations: 2 μ sections were cut from biopsy specimens after fixation in formalin–alcohol and embedding in paraffin. Masson trichrome, Jones, hematoxylin–eosin, and periodic acid–Schiff staining were used on the sections. The renal biopsies were classified according to the total inflammation score and the interstitial fibrosis/tubular atrophy score calculated using the Banff classification.^[25,26]^ The renal biopsies were classified according to the total inflammation score (ti score) and the interstitial fibrosis/tubular atrophy score calculated using the Banff classification. The ti score used semiquantitative criteria to assess the infiltration of the renal interstitial parenchyma by inflammatory cells. The severity was graded as no significant interstitial inflammation (<10% of parenchyma, grade 0), mild interstitial inflammation (10–25% of parenchyma, grade I), moderate interstitial inflammation (26–50% of parenchyma, grade II), and severe interstitial inflammation (>50% of parenchyma, grade III). Interstitial fibrosis and tubular atrophy were combined into 1 score: severity was graded by tubulointerstitial features as no significant interstitial fibrosis and tubular atrophy (grade 0), mild interstitial fibrosis and tubular atrophy (<25% of cortical area, grade I), moderate interstitial fibrosis and tubular atrophy (26–50% of cortical area, grade II), and severe interstitial fibrosis and tubular atrophy (>50% of cortical area, grade III).

For immunofluorescence-labeling evaluations, sections were cut from frozen biopsy specimens. Then, antiserum against C1q, C3, IgA, IgM, IgG, light Kappa, and Lambda chains were used.

### 2.3 Follow-up

Data regarding renal function (SCrl, eGFR, and need of dialysis), ophthalmic lesions were collected at 1 year when available (n = 35) and at the end of the follow-up in all patients (n = 41). The annual number of attacks was calculated as the ratio between the number of attacks since the 1st symptoms to the duration of follow-up in years (in patients followed for more than 6 months).

During the follow-up, treatment (methylprednisolone pulses, oral corticosteroids, local ophthalmic treatments), renal, or ophthalmologic relapses were recorded. A renal or an ophthalmologic relapse was defined as the reappearance of symptoms after a symptom-free period of at least 2 weeks. Patients were categorized into 3 groups according to the initial dose of oral corticosteroids: the “High dose” group, which received an initial dose of 1 mg/kg per d, the “intermediate dose” group, which received 0.5 to 0.7 mg/kg per d and the “no corticosteroids” group.

### 2.4 Statistical analysis

Continuous variables were described as medians and ranges and categorical variables as numbers and percentages. Quantitative demographic data were compared using the nonparametric Mann–Whitney test or Kruskal–Wallis test, when appropriate. Qualitative data were compared using Fisher exact test or χ^2^, when appropriate. The association between 2 quantitative variables was tested with a Spearman or Pearson correlation according to the distribution. The association between 1-year eGFR and explicative variables was studied by means of 2 linear regression models for each explicative variable: Model 1 consisted of a simple regression model with the follow-up eGFR as the dependent variable, whereas model 2 was a mixed model that took into account the center effect as a random effect and adjusted for the peak eGFR value. The effect of the interaction between treatment and peak creatinine and treatment choice was tested in both models and dropped if nonsignificant. For these models, multiple imputations of missing data were conducted, if needed. Two-tailed *P* < 0.05 was considered statistically significant. All statistical tests were computed using SAS 9.4 (Cary, NC) and GraphPad Prism version 6.00 (GraphPad Software, La Jolla, CA).

## 3 Results

### 3.1 Baseline characteristics

**Figure 1:**
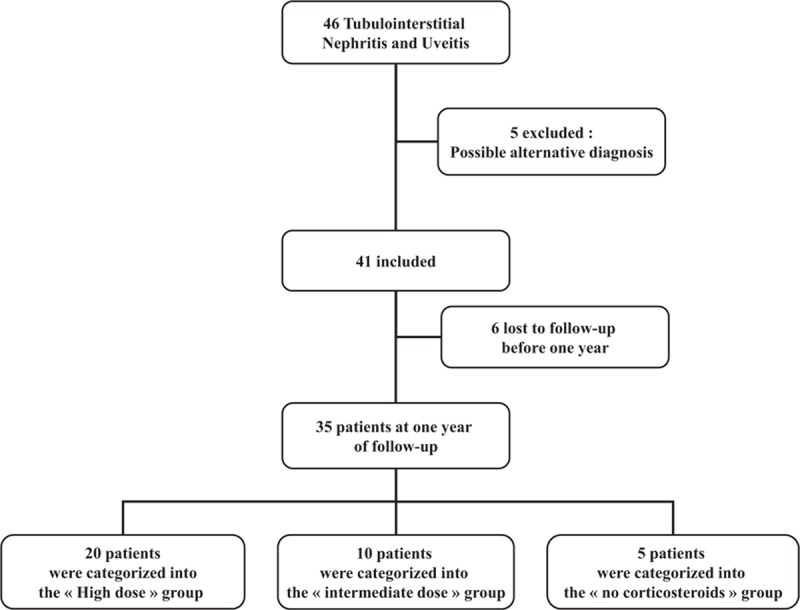
Flow Chart of patients’ corticosteroid treatment.

Altogether, 41 cases (25 females and 16 males: F/M ratio: 1.6:1) of TINU syndrome were identified (Fig. 1). Table 1 summarizes the patients’ main clinical and biological characteristics. The median age at disease onset was 46.8 years (range 16.8–77.4), and age at diagnosis was 47.0 years (range 16.9–77.4). The median age at disease onset was higher in women than in men (50.0 vs 40.8 years), though the difference was not significant. No patient had a family history of TINU syndrome. A single patient (patient no. 3) suffered from chronic kidney disease before the TINU with steady serum creatinine at 120 μmol/L (eGFR at 58 mL/min per 1.73 m^2^). Other major medical histories are described in Table 2.

**Table 1:**
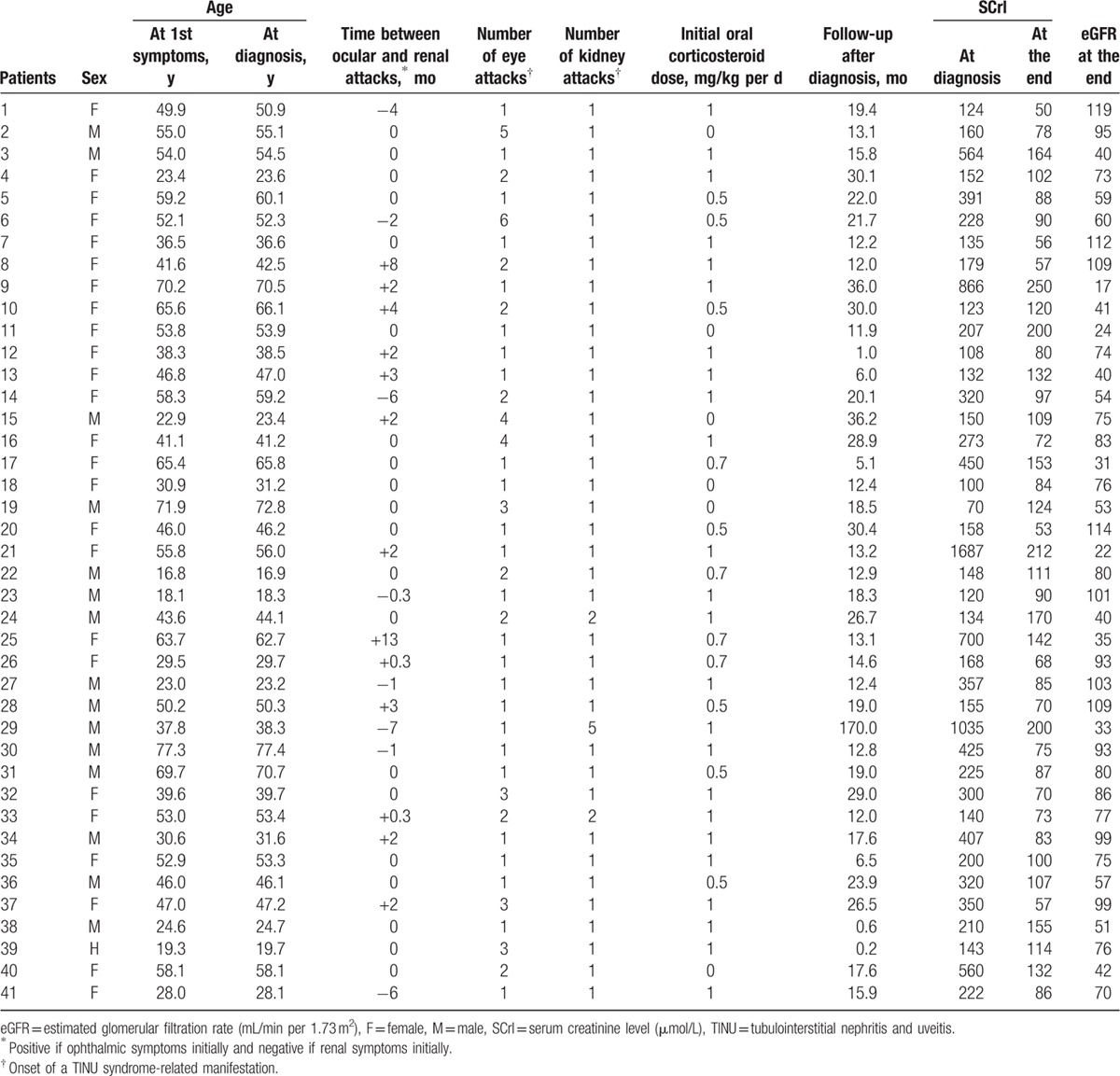
Patients’ characteristics and evolution.

**Table 2:**
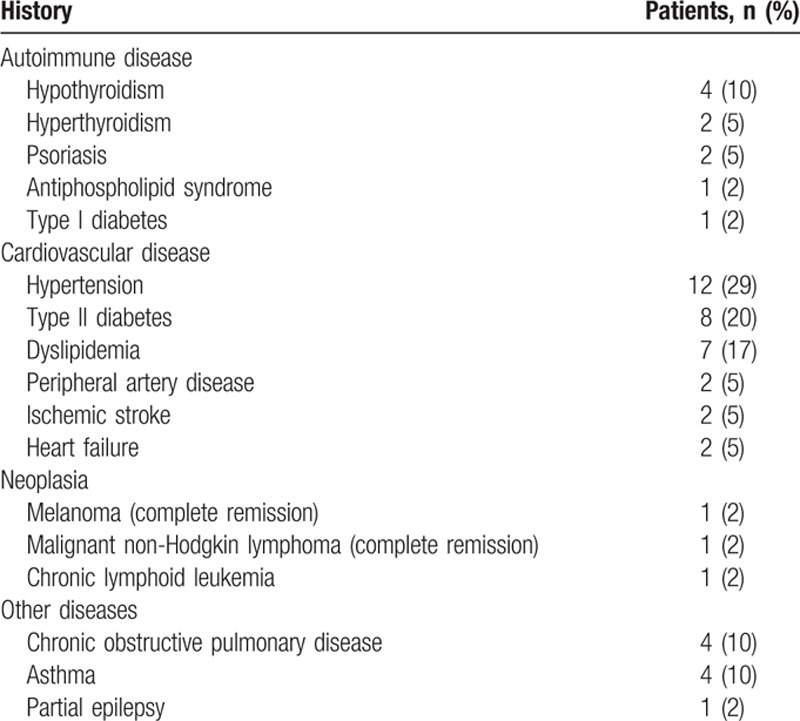
Principal medical history of patients.

Twenty-eight patients had «definite» and 13 patients had «probable» TINU syndrome. At disease-onset, renal symptoms preceded the uveitis in 8 patients (20%), with a median of 3.0 months (range 0.3–7.0) between the 2 attacks. On the contrary, the uveitis preceded the renal symptoms in 13 patients (31%), with a median of 2 months (range 0.3–13.0). In the 20 remaining patients (49%), the eye and kidney symptoms were concomitant. Four patients (10%) with initially severe renal symptoms underwent temporary hemodialysis (patients no. 9, 21, 25, and 29). General and eye symptoms at disease onset are summarized in Table 3.

**Table 3:**
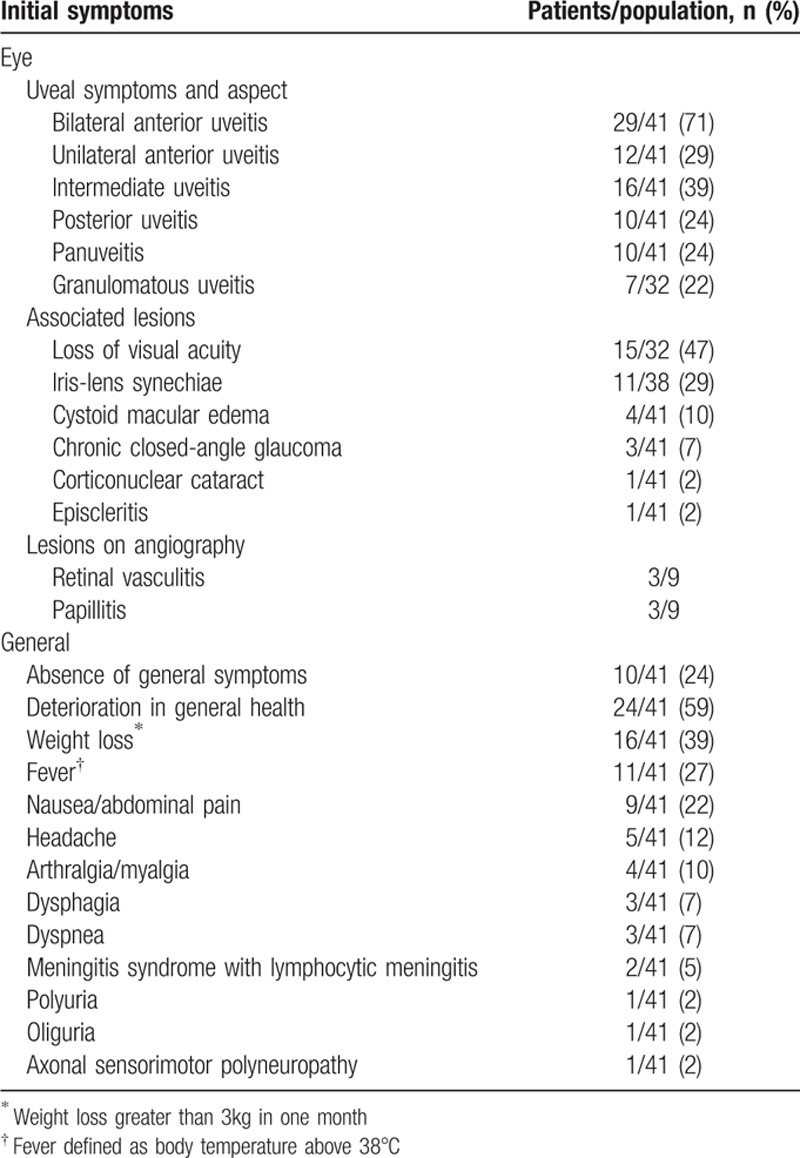
Symptoms at the 1st attack.

All patients had a rise in the SCrl, with median SCrl at the 1st flare of 207 μmol/L (range 100–1687) and a median eGFR of 27 mL/min per 1.73 m^2^ (range 2–73). Proteinuria was above 0.3 g/24 h in 32 patients (78%), with median proteinuria of 0.52 g/24 h (range 0.10–2.10). Thirty-one patients (76%) had a concomitant inflammatory syndrome, with a median CRP level of 16 mg/L (range 0–217). Twenty-one out of 40 patients (53%) had a median hemoglobin level of 10.6 g/dL (range 6.7–15.9); and 3 patients presented with blood eosinophilia at 0.6, 1.0, and 1.2 g/L. We found median levels of serum calcium at 2.25 mmol/L (range 2.05–2.53), phosphoremia at 1.02 mmol/L (range 0.63–2.54), plasma bicarbonate at 22 mmol/L (range 8–31), and albuminemia at 39.5 g/L (range 25–45). Polyclonal hypergammaglobulinemia was found in 17 of 35 patients (49%), with a median gammaglobulin level of 13.1 g/L (range 11.3–28.2). The abnormal urinary findings at inclusion are described in Table 4.

**Table 4:**
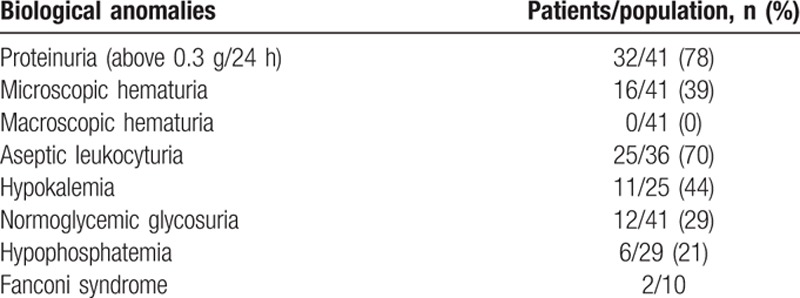
Abnormal urinary and biological findings at the 1st attack.

### 3.2 Pathologic renal findings

**Figure 2:**
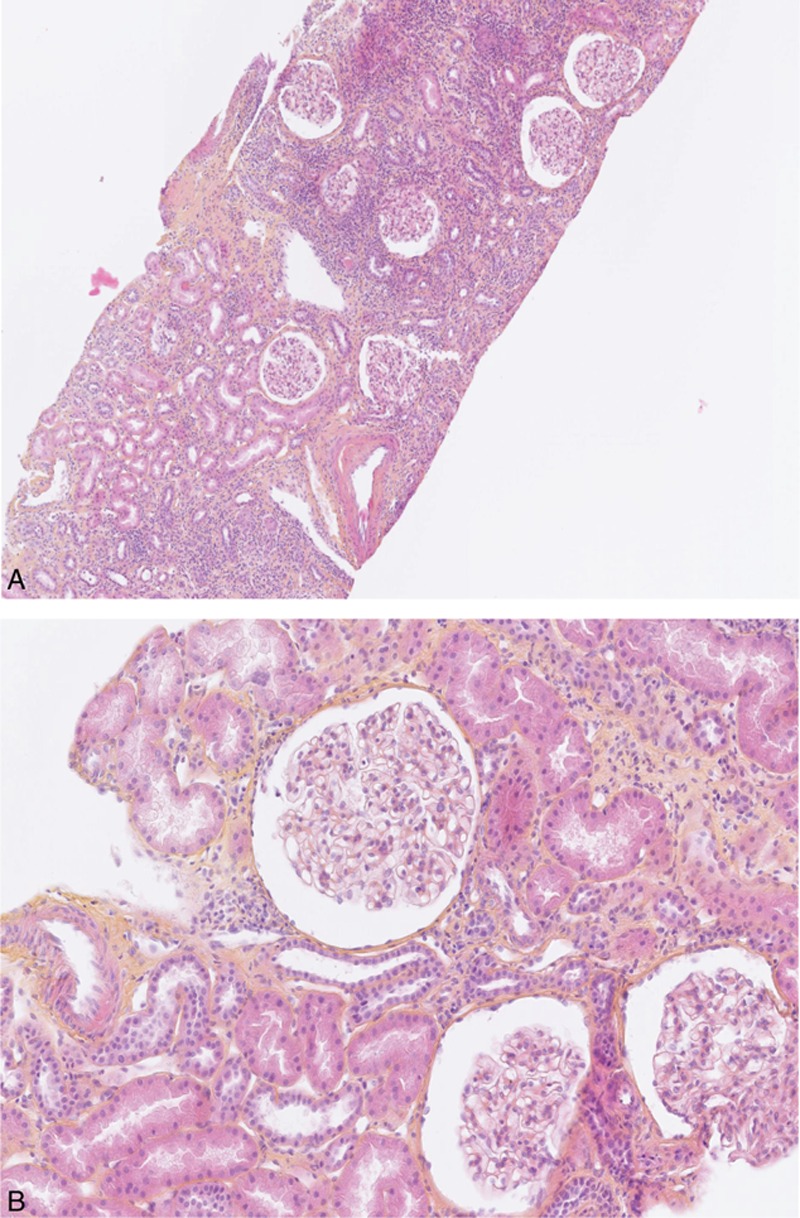
Kidney lesions of TINU syndrome. (A) Low-power view showing diffuse interstitial inflammation composed of mononuclear cells, lesions of interstitial fibrosis and tubular atrophy, and no glomerular or vascular lesions (hematoxylin and eosin, magnification ×200). (B) Normal glomeruli (hematoxylin and eosin, magnification ×400). TINU = tubulointerstitial nephritis and uveitis.

All renal biopsies showed acute tubulointerstitial nephritis (Fig. 2). The ti score (n = 37) revealed: 4 patients with grade I (11%), 4 patients with grade II (11%), and 29 patients with grade III (78%). Among the inflammatory cells infiltrating the renal interstitium, there were associations of lymphocytes (n = 23/23; 100%), plasmocytes (n = 13/23; 57%), polynuclear neutrophils (n = 7/23; 30%), polynuclear eosinophils (n = 7/24; 29%), and histiocytes (n = 6/23; 26%). The evaluation of the interstitial fibrosis and tubular atrophy revealed: 20 patients with grade 0 (51%), 11 patients with grade I (28%), and 8 patients with grade II (21%). Acute tubular necrosis was found in the renal biopsies of patients no. 3, 9, 35, 36, and 37 (12%). Signs of glomerular ischemia were present in 6 biopsies (Patients no. 7, 9, 10, 28, 30, and 31) (15%). Focal thickening of the glomerular mesangium was found in 1 patient (patient no. 22). Immunofluorescence showed mild isolated mesangial C3 deposits in patients no. 33 and 39 and mild combined IgA, IgM, and C3 focal segmental deposits in patients no. 24 and 41. Lesions of mild arteriosclerosis were found in 4 patients (patients no. 6, 10, 17, and 27) (10%).

### 3.3 Treatments

At the diagnosis of TINU syndrome, the renal involvement in 35 patients was treated with oral corticosteroids (85%). None of the 6 remaining patients were given any systemic treatment during their follow-up (patients no. 2, 11, 15, 18, 19, and 40). The median duration of the systemic corticotherapy was 8.0 months (range 0.2–39.0). Seven patients were given 3 initial pulses of methylprednisolone before the start at 1 mg/kg per d (patients no. 3, 9, 21, 22, and 24) or at 0.5 to 0.7 mg/kg per d (patients no. 16 and 36) of oral corticosteroids. In patients treated with oral corticosteroids, the median dose was 1 mg/kg per d (range 0.5–1.0). In 2 patients, recurrent TINU syndrome was treated with a 2nd course of systemic corticotherapy. No patient was treated with other immunosuppressive drug.

Data regarding the initial ophthalmic treatment were available for 34 patients. All of these patients were treated with cortisone eye drops. Ophthalmic recurrences were treated with topical corticoids alone (n = 8), systemic corticotherapy (n = 4) with methylprednisolone pulses (n = 2), subtenon injection of triamcinolone acetonide (n = 2), and colchicine (n = 1).

### 3.4 Outcomes

The median duration of follow-up after diagnosis was 17.8 months (range 0.2–170.0). At the end of follow-up, the evaluation of renal function showed a median eGFR of 75 mL/min per 1.73 m^2^ (range 17–119) and a median SCrl of 90 μmol/L (range 50–250). No patient was on dialysis. The median number of attacks per patient per year was 1.0 (range 0.1–4.6) for eye symptoms and 0.8 (range 0.2–2.0) for renal symptoms.

**Figure 3:**
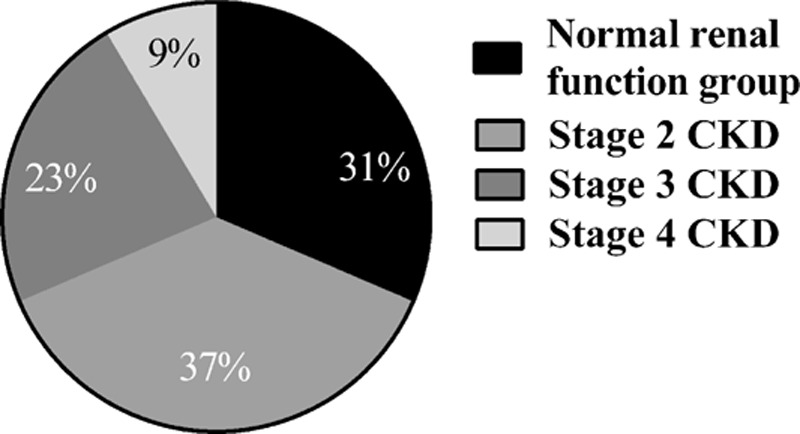
Evaluation of renal function at 1 y of follow-up in 35 patients with TINU syndrome. (A) Renal prognosis after 1 y of follow-up. The eGFR in the normal renal function group calculated using the MDRD was above 90 mL/min per 1.73 m^2^. The eGFR in the group with stage 2 CKD was between 60 and 90 mL/min per 1.73 m^2^. The eGFR in the group with stage 3 CKD was between 30 and 60 mL/min per 1.73 m^2^. The eGFR in the group with stage 4 CKD ranged from 15 to 30 mL/min per 1.73 m^2^. CKD = chronic kidney disease, eGFR = estimated glomerular filtration rate, MDRD = modification of diet in renal disease, TINU = tubulointerstitial nephritis and uveitis.

Factors associated with renal and ophthalmologic outcomes were studied among patients with at least 1 year of follow-up (n = 35). Among these patients, at 1 year, the median eGFR was 76 mL/min per 1.73 m^2^ (range 17–119), and the median SCrl was 87 μmol/L (range 50–250). The renal prognosis at 1 year of follow-up is shown in Fig. 3. Nine percent (n = 3/35) of these patients had relapses of acute renal insufficiency. Twenty patients received high-dose corticosteroids (i.e., initial dose of 1 mg/kg per d), with median duration of treatment of 12 months (range 3–12), 10 patients received an intermediate dose (i.e., 0.5–0.7 mg/kg per d) initial with a median duration of treatment of 8 months (range 2–12), and 5 patients were not given systemic corticotherapy.

At the 1st renal flare, there was no significant difference between the 3 treatment-regimen groups regarding age (*P* = 0.24) and SCrl (median SCrl [range] in μmol/L: 260 [108–1687], 226 [123–700], and 160 [100–360] in the high dose, intermediate dose, and untreated groups, respectively) (*P* = 0.61), eGFR (median eGFR [range] in mL/min per 1.73 m^2^: 22 [2–73], 24 [5–44], and 42 [12–60] in the high, intermediate, and untreated group, respectively) (*P* = 0.20). At 1 year of follow-up, no difference appeared between those groups regarding SCrl (median creatinine [range] in μmol/L: 87 [50–150], 82 [50–142], and 111 [78–192] in high, intermediate, and untreated group, respectively) (*P* = 0.44) and eGFR (median eGFR [range] in mL/min per 1.73 m^2^: 79 [17–119], 77 [35–114], and 75 [24–95] in the high, intermediate, and untreated group, respectively) (*P* = 0.65).

There was no significant difference between the 3 treatment-regimen groups regarding age and serum creatinine, eGFR at the 1st renal flare or eGFR and creatinine at 1 year of follow-up (*P* = 0.24, 0.61, 0.20, 0.65, and 0.44, respectively). Nonetheless, the percentage of improvement in SCrl at 1 year of follow-up was significantly greater in the “intermediate dose” group than in “no corticosteroids” group (*P* = 0.04). The difference between the “high-dose” group and the “no corticosteroids” group was borderline significant (*P* = 0.06) (Fig. 3B). Taken together, patients receiving corticosteroids (n = 30) had a greater percentage of improvement in SCrl and eGFR than those without corticosteroids (*P* = 0.02 and 0.03, respectively).

Using univariate linear regression, the following variables were significantly associated with 1-year follow-up eGFR: SCrl (*P* value, linear regression coefficient and 95% confidence interval (β), correlation coefficient (r) were, respectively, *P* < 0.001, β = −0.1 [−0.1; −0.0], r = −0.54), serum bicarbonate levels (*P* = 0.01, β = 3[0.8; 5.3], r = 0.53), serum phosphate levels (*P* = 0.04, β = −27.2 [−51.3; −3.0], r = 0.46), and age (*P* = 0.03, β = −0.6 [−1.2; −0.0], r = −0.37) at the 1st symptoms. Using mixed models adjusted for peak eGFR and center effect resulted in a loss of significance of serum bicarbonate level (*P* = 0.21, β = 1.6 [−1.1; 4.4]), serum phosphate level (*P* = 0.09, β = −24.4 [−53.3; 4.4]), and age (*P* = 0.24, β = −0.4 [−1.1; 0.3]). The effect of treatment^∗^peak eGFR was not significant in any model and was thus excluded from final models. No association was found between eGFR at 1 year and initial proteinuria (*P* = 0.71, β = 3.3 [−17.2; 23.8], r = 0.07), CRP (*P* = 0.77, β = 0.0 [−0.3; 0.2], r = 0.05), gammaglobulinemia levels (*P* = 0.93, β = 1.2 [−1.5; 4.0], r = −0.02), or the time between renal and uveal involvement (*P* = 0.92, β = 0.00 [−0.3; 0.3], r = 0.00) or the number of recurrences of uveitis at 1 year (*P* = 0.53, β = −1.6 [−10.8; 7.5], r = −0.11) in simple linear regression. Neither were these variables associated with 1-year eGFR after adjusting for peak eGFR and center effect using mixed models. The eGFR at 1 year in patients who presented aseptic leukocyturia or thyroid abnormalities was not significantly different from that in patients without these conditions (*P* = 0.71 and 0.51, respectively). The type of initial uveal symptoms was not a predictor of the eGFR after 1 year of follow-up (data not shown).

As for pathological findings, patients who had grade III total inflammation had a worse eGFR (median 23 mL/min per 1.73 m^2^ [range 4–58]) than those with ti grade II (median 53 mL/min per 1.73 m^2^ [range 40–73]) (*P* = 0.03). Regarding the initial SCrl, this difference was borderline significant (*P* = 0.06). Nevertheless, the initial eGFR (and initial SCrl) were not different between patients with grade I (median 34 mL/min per 1.73 m^2^ [range 2–60]) and grade II total inflammation and between those with grades I and III total inflammation. After 1 year of follow-up, the eGFR (or the SCrl) was not significantly different between these 3 grade groups (with median eGFR of 76 mL/min per 1.73 m^2^ [range 22–99], 75 mL/min per 1.73 m^2^ [range 41–90], and 74 75 mL/min per 1.73 m^2^ [range 17–119], respectively, for grades I, II, and III). There was no significant difference between the 3 treatment-regimen groups regarding the initial ti grade (*P* = 0.26). Neither the grade of tubular atrophy and cortical fibrosis, nor the type of cells infiltrating the renal interstitium, nor the vascular injuries, nor the glomerular injuries was associated with eGFR decrease. Initial eGFR was significantly lower among patients who presented acute tubular necrosis (*P* = 0.03), but after 1 year of follow-up, there was no significant difference (*P* = 0.09 and 0.13, respectively).

During follow-up, 4 chronic closed-angle glaucoma, 1 neovascular glaucoma, 2 corticonuclear cataracts, and 1 ocular ischemia syndrome were noted. At the end of the follow-up, 3 patients had chronic anterior uveitis and 2 patients presented chronic panuveitis.

**Figure 4:**
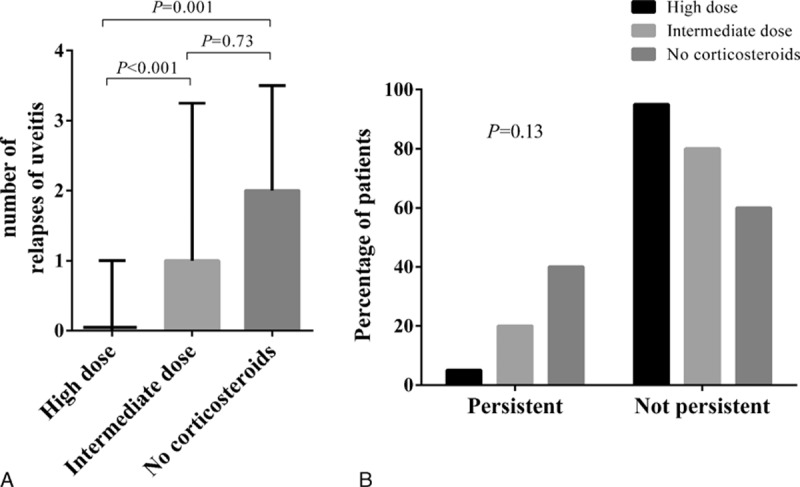
Evaluation of the ophthalmological prognosis after 1 y of follow-up according to the dose of oral corticosteroids initially administered in 35 patients with TINU syndrome. Group A corresponds to patients who received oral corticosteroids at 1 mg/kg per d (n = 19); Group B a dose between 0.5 and 0.7 mg/kg per d (n = 10); Group C had no systemic treatment (n = 4). The statistical differences (*P*) between the groups are shown. (A) Evaluation of the number of relapses of uveitis related to TINU syndrome. The bars correspond to medians and the whiskers to the interquartile range. (B) Evaluation of persistent chronic uveitis related to TINU syndrome. The bars correspond to the percentage of patients. TINU = tubulointerstitial nephritis and uveitis.

After 1 year of follow-up, 46% (n = 16/35) of the patients had uveitis relapses (median number of relapses 0 [range 0–4]). Patients receiving high-dose corticosteroids had significantly fewer relapses of uveitis after 1 year than did patients receiving intermediate-dose or no corticosteroids (*P* < 0.001 and *P* = 0.001, respectively). The number of relapses of uveitis was not significantly different between the “intermediate-dose” and “no corticosteroids” groups (*P* = 0.73) (Fig. 4A). Taken together, patients receiving corticosteroids had fewer relapses of uveitis than did those without corticosteroids (*P* = 0.02). After adjusting for peak eGFR and taking into account the center effect in a mixed model, the treatment effect remained globally significant (*P* = 0.02). Taking the “no corticoid group as reference,” the number of relapses was significantly lower in the “high-dose” group (*P* = 0.01), while it was not significant in the “intermediate-dose” group (*P* = 0.13).

At 1 year, 4 patients had signs of chronic anterior uveitis and 1 patient had chronic panuveitis with cystoid macular edema (patients no. 4, 6, 15, 16, and 18) (14%). The distribution of patients with persistent uveitis at 1 year, according to the initial systemic treatment with corticosteroids is shown in Fig. 4B. A subtenon injection of triaminolone acetonide in patients no. 14 and 15 resulted in a significant improvement in local symptoms (completely resolved in <3 months in patient no. 14). Both these patients had just experienced a recurrence of their uveitis after stopping the systemic corticotherapy.

## 4 Discussion

TINU syndrome is a rare disease with a brutal onset and is characterized by ocular and renal involvement.^[2]^ Here, we report on the largest series of adult patients to date and describe the clinical, biological, and pathological findings, as well as treatment regimens and medium-term prognosis. Despite the retrospective design of this study, several pertinent observations can be made.

First, the clinical and biological characteristics of our patients are in accordance with previously reported cases.^[2,6,15]^ As expected, we found a predominance of females (F/M ratio 1.6:1) with a median age at disease onset (46.8 years) close to that reported by Li et al in 2014 (47.7 years).^[2,6,15,21,22]^ Whereas eye symptoms tended to recur (40% of patients at 1 year of follow-up), renal symptoms rarely recurred (9% of patients). In contrast, progression to chronic disease was rare for eye symptoms (14% of patients at 1 year) and more frequent for renal symptoms (69% at 1 year). These findings had already been reported, but the lack of systematic follow-up with a standardized form makes comparisons difficult.^[2,6,15]^ Interestingly, at disease onset, 2 patients were diagnosed with lymphocytic meningitis, which has never been reported before. In these 2 patients, there was no evidence for an alternative diagnosis other than a TINU syndrome. The meningitis resolved with no neurological sequelae. Our cohort also adds 2 new cases of TINU syndrome-related Fanconi syndrome (patients no. 4 and 20) to the 5 previously published.^[7,24,27,28]^

While TINU syndrome is usually described as having a favorable renal outcome,^[2,6,22,29]^ our data and that of others^[15]^ suggest that full renal recovery is not the norm. According to Li et al, this difference between older data in the literature and more recent results could also be explained by the fact that in the majority of articles the medium- and long-term follow-up was not described, and that renal function was expressed as serum creatinine and not eGFR.^[15]^ However, whereas Li et al reported an eGFR below 60 mL/min per 1.73 m^2^ after 1 year of follow-up in 80% of their patients, we found a rate of 32%. This difference cannot be explained by differences in age, medical history, or initial increases in SCrl between the 2 cohorts, as both were comparable for these criteria. The patients described by Li et al were systematically treated with corticosteroids associated with cyclophosphamide in cases of renal dysfunction requiring hemodialysis or relapses of acute increases in SCrl. In our study, only corticosteroids were used, and not systematically. Even if our 2 studies differed with regard to the therapeutic management, it seems unlikely that the differences between the 2 groups of patients for renal prognosis could have been related to the treatment. Disease severity could be at least in part predetermined by the patients’ genetic background, and it could be hypothesized that Chinese patients could be more likely to develop sequelae. Many of the patients in our cohort were lost to follow-up and did not benefit from long-term care despite having chronic kidney disease. Given the impaired renal function in the medium term, patients suffering from TINU syndrome should benefit from long-term care, including the prevention of cardiovascular risk and the management of complications of chronic kidney disease.

This work aimed to bring to light several factors associated with long-term renal prognosis. First, the older the patient, the poorer was the renal prognosis after 1 year of follow-up. This difference could in part be explained by the tendency for accelerated fibrosis in the older patients.^[30]^ Second, the initial severity of renal symptoms was associated with renal function after 1 year of follow-up. The higher the initial SCrl, the lower the GFR after 1 year of follow-up. This work showed that the initial serum bicarbonate and phosphate levels were associated with eGFR after 1 year of follow-up. The abnormal levels of these biological markers probably reflected the severity of the acute renal insufficiency. Indeed, in multivariate analyses only the initial peak serum creatinine was associated with eGFR after 1 year of follow-up. Contrary to what was previously reported, the presence of leukocyturia and a history of auto-immune thyroiditis were not associated with the fall in the eGFR.^[15]^ Last, as with other studies, we found no correlation between the severity of eye symptoms and that of renal symptoms.^[2,6,15]^

To date, no pathological markers have proved to be prognostic factors in TINU syndrome.^[2,15]^ A large majority of patients had severe interstitial inflammation. Severe interstitial inflammation seemed to be associated with a lower initial eGFR. Thus, this difference was only noted between patients who had severe inflammation and those who had moderate inflammation and it disappeared after 1 year of follow-up. Maybe a significant difference between severe and mild inflammation could have been noted in a bigger cohort. In this study, patients who had acute tubular necrosis had a greater deterioration in kidney function than did patients without such lesions. However, after 1 year of follow up, these patients shared the same kidney outcome. Nonetheless, the initial severity of these lesions was not known, and severe acute tubular necrosis might induce renal sequelae. The grade of interstitial fibrosis and renal cortical tubular atrophy were not prognostic factors for renal outcomes in this work. In certain patients, it is possible that the grade of interstitial fibrosis was overestimated (due to local inflammation).^[31]^ However, in transplantation medicine, it is believed that the extent of the underlying disease, rather than the interstitial fibrosis and renal cortical tubular atrophy, is linked to a worse outcome.^[26]^ As for immunofluorescence, 1 diabetic patient presented mild mesangial C3 deposits without visible glomerular lesions. These deposits could reflect an early stage of diabetic glomerulosclerosis or could be nonspecific.^[32]^ An association of glomerular IgA, IgM, and C3 deposits without mesangial thickening or proliferation was found in 2 biopsies. There were no arguments for IgA vasculitis or cirrhosis-associated nephropathy. These patients seemed to have latent mesangial IgA deposition, as seen in 4% to 16% of «healthy» individuals.^[33]^ Three patients had vascular lesions associated with hypertension, and another 1 associated with diabetes. In this work, the renal prognosis was no different in patients with or without glomerular and/or vascular lesions. Our study was based on retrospective data in a small cohort and the renal biopsy was interpreted by several different assessors. Hence, it remains open to question whether a standardized evaluation of the fibrosis grade and the inflammatory infiltrate could be prognostic markers of a decrease in the eGFR in TINU syndrome.

As TINU syndrome is a very rare disease, treatment is not standardized. The treatment choice was up to clinicians in each center. This was taken into account in multivariate models by the means of mixed model with center effect as a random effect. In this study, we could not identify factors that determined clinicians’ choices. Neither the initial peak SCrl, nor the age, nor the pathological findings seemed to be related to treatment choice. Nonetheless, even though it was not significant, the peak SCrl at the 1st flare was not as high among untreated patients as among treated patients.

After 1 year of follow-up, eGFR was not statistically different between patients treated with or without corticosteroids. Nonetheless, the percentage variations in SCrls and eGFR showed a greater improvement in patients treated with corticosteroids. The serum creatinine decrease was greater in patients treated with an initial dose of 0.5 to 0.7 mg/kg per d than in untreated patients. The improvement in renal function associated with corticosteroids has only been reported in isolated cases.^[2,34]^ However, even though we report here on the largest ever published cohort of patients with TINU syndrome, our results should be extrapolated with caution. The number of patients is still small (the subgroup of untreated patients included only 5 patients after 1 year of follow-up). Hence, the difference in the eGFR after 1 year between the group given 1 mg/kg per d and the untreated group was borderline significant. This difference improvement might have been significant in a study involving a bigger sample size. Indeed, the patients who were untreated had the best initial peak serum creatinine but the worst outcome, thus it not was not significant. The interest of oral corticosteroids in the treatment of renal symptoms needs to be evaluated in a prospective study. One of the other biases could have come from the fact that some patients were treated with a bolus of methylprednisolone (none from the untreated group).

Treatment with oral corticosteroids was associated with an improvement in the uveal symptoms. All 5 patients who were not treated with oral corticosteroids suffered from 1 to 4 recurrences of their uveitis during the 1st year of follow-up. Patients treated with 1 mg/kg per d had statistically fewer recurrences (13/20 had no recurrence). These new results are in agreement with the few data presented in the literature.^[6]^ The trend toward a lower frequency of persistent uveitis in patients treated with corticosteroids was borderline significant. Two patients from the 1 mg/kg per d group with chronic uveitis experienced a significant improvement in their eye symptoms after a subtenon injection of triamcinolone acetonide. The interest of oral corticosteroids in the treatment of recurrent uveitis is tempered by these data (only for the 1 mg/kg per d group). Other immunomodulatory and/or immunosuppressant treatments (i.e., azathioprine, ciclosporine, mycophenolate mofetil, or methotrexate) have anecdotally been reported as successful treatments for eye symptoms.^[2,7,35–37]^ In light of recent data suggesting the possible role of humoral immunity (i.e., of anti-mCRP antibodies) in the pathogenesis of TINU syndrome, other therapeutic strategies targeting B-lymphocytes warrant further evaluation.^[8,15]^

While our study has several strengths, there are a number of limitations. Some data were inevitably missing and some patients were lost to follow-up due to the retrospective study design. Next, since no serum had been kept frozen for further analysis, we were also unable to measure circulating levels of mCRP, which have previously been suggested as a potential biomarker of late-onset uveitis flares.^[14,15]^

In conclusion, 32% of patients were suffering from moderate to severe chronic kidney disease after 1 year of follow-up, and 40% had uveitis relapses during this follow-up. High initial serum creatinine was associated with a low eGFR at 1 year. Oral corticosteroids were associated with a smaller number of recurrences of uveitis but did not seem to improve the kidney function.
